# Utility of silhouette showcards to assess adiposity in three countries across the epidemiological transition

**DOI:** 10.1371/journal.pgph.0000127

**Published:** 2022-05-19

**Authors:** Tyler O. Reese, Pascal Bovet, Candice Choo-Kang, Kweku Bedu-Addo, Terrence Forrester, Jack A. Gilbert, Julia H. Goedecke, Estelle V. Lambert, Brian T. Layden, Lisa K. Micklesfield, Jacob Plange-Rhule, Dale Rae, Bharathi Viswanathan, Amy Luke, Lara R. Dugas

**Affiliations:** 1 Stritch School of Medicine, Loyola University Chicago, Maywood, Illinois, United States of America; 2 Department of Medicine, University of Colorado School of Medicine, Aurora, Colorado, United States of America; 3 Unit for Prevention and Control of Cardiovascular Disease, Ministry of Health, Victoria, Republic of Seychelles; 4 University Center for Primary Care and Public Health, Lausanne, Switzerland; 5 Parkinson School of Health Sciences and Public Health, Loyola University Chicago, Maywood, Illinois, United States of America; 6 Department of Physiology, Kwame Nkrumah University of Science and Technology, Kumasi, Ghana; 7 Solutions for Developing Countries, University of the West Indies, Mona, Kingston, Jamaica; 8 Department of Surgery, Microbiome Center, University of Chicago, Chicago, Illinois, United States of America; 9 Division of Exercise Science and Sports Medicine, Department of Human Biology, Research Centre for Health through Physical Activity, Lifestyle and Sport, Faculty of Health Sciences, University of Cape Town, Cape Town, South Africa; 10 Non-Communicable Diseases Research Unit, South African Medical Research Council, Cape Town, South Africa; 11 Division of Endocrinology, Diabetes, and Metabolism, University of Illinois at Chicago, Chicago, Illinois, United States of America; 12 Jesse Brown Veterans Affairs Medical Center, Chicago, Illinois, United States of America; 13 SAMRC/Wits Developmental Pathways for Health Research Unit, Department of Pediatrics, Faculty of Health Sciences, University of the Witwatersrand, Johannesburg, South Africa; Public Health Foundation of India, INDIA

## Abstract

The Pulvers’ silhouette showcards provide a non-invasive and easy-to-use way of assessing an individual’s body size perception using nine silhouette shapes. However, their utility across different populations has not been examined. This study aimed to assess: 1) the relationship between silhouette perception and measured anthropometrics, i.e., body mass index (BMI), waist circumference (WC), waist-height-ratio (WHtR), and 2) the ability to predict with silhouette showcards anthropometric adiposity measures, i.e., overweight and obesity (BMI ≥ 25 kg/m^2^), obesity alone (BMI ≥ 30 kg/m^2^), elevated WC (men ≥ 94 cm; women ≥ 80 cm), and WHtR (> 0.5) across the epidemiological transition. 751 African-origin participants, aged 20–68 years old, from the United States (US), Seychelles, and Ghana, completed anthropometrics and selected silhouettes corresponding to their perceived body size. Silhouette performance to anthropometrics was examined using a least-squares linear regression model. A receiver operator curve (ROC) was used to investigate the showcards ability to predict anthropometric adiposity measures. The relationship between silhouette ranking and BMI were similar between sexes of the same country but differed between countries: 3.65 [95% CI: 3.34–3.97] BMI units/silhouette unit in the US, 3.23 [2.93–3.74] in Seychelles, and 1.99 [1.72–2.26] in Ghana. Different silhouette cutoffs predicted obesity differently in the three countries. For example, a silhouette ≥ five had a sensitivity/specificity of 77.3%/90.6% to predict BMI ≥ 25 kg/m^2^ in the US, but 77.8%/85.9% in Seychelles and 84.9%/71.4% in Ghana. Ultimately, silhouettes predicted BMI, WC, and WHtR similarly within each country and sex but not across countries. Our data suggest that Pulvers’ silhouette showcards may be a helpful tool to predict anthropometric and adiposity measures in different populations when direct measurement cannot be performed. However, no universal silhouette cutoff can be used for detecting overweight or obesity status, and population-specific differences may stress the need to calibrate silhouette showcards when using them as a survey tool in different countries.

## Introduction

The prevalence of overweight and obesity is increasing in populations spanning the epidemiological transition and may be particularly high in individuals of African-origin [1–4]. In addition, elevated weight has been associated with the development of non-communicable diseases (NCDs) [5–8]. To assess for obesity, body mass index (BMI, kg/m^2^) is widely used because of its simplicity and ease of measurement. However, BMI does not discriminate well between adipose and lean mass. Therefore, waist circumference (WC) and waist-to-height ratio (WHtR) have been suggested to predict adiposity better and have been shown to correlate well with fat mass as assessed by accurate methods such as computed tomography (CT) [9–12].

Measures of adiposity that do not rely on actual measurements may be helpful in some situations. Examples include surveys and studies in public health, anthropology, economics, and marketing that must be performed without direct contact with a respondent (e.g., mail-order or internet-based) or situations to avoid the burden of asking participants to remove clothing. Furthermore, self-reported adiposity (e.g., height and weight) is prone to reporting bias, depends on access to anthropometric tools like scales, and can be influenced by cultural views on body size [13–19].

**Fig 1:**
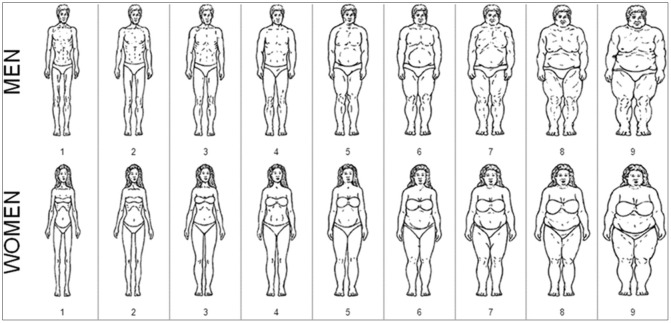
Pulvers’ silhouettes designed for populations of African-origin. Source: Pulvers 2004, Obesity Res.

Initially developed by Stunkard and colleagues, sex-specific silhouette showcards (referred to as “silhouettes” hereafter) can be used to determine one’s perception of their body size. This tool relies on presenting a series of drawings of distinct body sizes in an increasing sequence. Respondents then select the silhouette they think best reflects their body size relative to objective measurements [20]. Silhouettes should be ethnically ambiguous enough to be used in different cultures but still detailed enough to be relatable. A variety of silhouette tools have been developed and validated for different populations [21–24]. For example, Pulvers and colleagues created culturally relevant silhouette showcards for African Americans (Fig 1) [25]. These silhouettes were validated in different populations of African-origin such as Seychelles, the Caribbean, and the US [25–28]. However, while many studies have shown a good association between the silhouettes and anthropometrics, including for the prediction of obesity, most studies have only assessed their validity in a single population at a time [21–32]. Also, no studies have directly compared the associations of silhouette ranking and anthropometrics between countries with different population mean BMI levels or stages of development. Thus, assessing the validity of silhouettes to predict adiposity in different populations may be challenging. As such, cross-cultural evaluation should rely on studies that use the same methodology in different countries [29, 33–36].

Therefore, our study aims to assess: 1) the relationship between silhouette perception and measured anthropometrics, i.e., body mass index (BMI), waist circumference (WC), waist-height-ratio (WHtR), and 2) the ability to predict with silhouette showcards anthropometric adiposity measures, i.e., overweight and obesity (BMI ≥ 25 kg/m^2^), obesity alone (BMI ≥ 30 kg/m^2^), elevated WC (men ≥ 94 cm; women ≥ 80 cm), and WHtR (> 0.5) in three African-origin populations with differing population mean BMI levels, as well as, stages of social and economic development.

## Methods

### Study populations and ethics approval

This study is a subset analysis from the METS-Microbiome study (R01-DK111848), for which the protocol has been published [37]. The first METS cohort consisted of 2,506 participants enrolled in December 2010 and January 2011, and 751 participants from this original cohort participated in this study. The METS-Microbiome study continues yearly measurements of participants initially recruited for the Modeling the Epidemiological Transition Study (METS; R01-DK080763). The METS-Microbiome study includes five African-origin populations spanning the epidemiologic transition varying by the United Nations Human Development Index (HDI) 2010. HDI is a statistical composite index of education, life expectancy, and per capita income indicators used to rank countries by human development [38, 39].

The current data presented was collected between 2018–2019 from participants in metropolitan Chicago, IL, US (HDI: 0.92), the mixed urban/rural Seychelles islands (0.80), and rural Ghana (0.59) [37, 39]. These three sites represent different social and economic development stages and have a largely different prevalence of obesity [39]. All participants were of African-origin except for Seychelles, where both African participants and participants of mixed ethnicity ancestry were included.

The METS-Microbiome protocol used in this study was approved by the Institutional Review Board of Loyola University Chicago, Chicago, IL, US (LU 209537), the National Research Ethics Committee of Seychelles, and the Committee of Human Research Publication and Ethics of Kwame Nkrumah University of Science and Technology, Kumasi, Ghana [37]. In addition, written informed consent was obtained from all participants.

### Survey and body size silhouette showcards

The survey component of the METS-Microbiome study consisted of a face-to-face interview performed by centrally trained personnel, capturing participants’ sociodemographic data, health-related behaviors, and medical history. Participants were also presented with sex and ethnicity-specific silhouette showcards created by Pulvers (Fig 1) [25, 27]. This nine-image tool displayed sex-specific body sizes in increasing order ranging from very thin to severely obese. Participant’s perceived body size was assessed by asking, “In the drawing, which figure best reflects how you think you look with regards to your body shape?”. Participants’ responses were recorded on a scale of 1 (representing the thinnest silhouette) to 9 (representing the most obese).

### Anthropometric and adiposity measurements

Anthropometric data, including measured height (m), weight (kg), and waist circumference (cm), was collected from each participant. Across all sites, standardized equipment and protocols were used, as previously published [37]. Body mass index (BMI, weight/height^2^) was calculated and classified as underweight (BMI < 18.5 kg/m^2^), normal weight (BMI 18.5–24.9 kg/m^2^), overweight (BMI 25.0–29.9 kg/m^2^) or obese (BMI ≥ 30 kg/m^2^) [40]. A dichotomous waist circumference (cm) variable was used to classify the presence of central obesity as defined by the International Diabetes Federation (≥ 94 cm in men, ≥ 80 cm in women) for European or African-origin individuals [11]. WHtR (waist in cm/ height in cm) was calculated and dichotomized using a widely used cut-off point for normal (WHtR ≤ 0.5) or increased central obesity (WHtR > 0.5) [41].

### Statistical analyses

Participant characteristics were summarized using means and 95% confidence intervals (CI). Proportions were calculated and presented as a percent (%) and 95% CI for categorical variables. Participant characteristics by sex were compared to the US using a two-sample t-test. In line with previous studies on this topic, Spearman’s rank correlation coefficients were used to describe the associations between the self-reported perceived silhouette ranking and BMI, WC, and WHtR.

Mean BMI and 95% CI for each silhouette rank were determined by sex and by country. To assess how the change in one silhouette ranking by anthropometric measures, such as BMI units per silhouette unit, differed between countries and sex, we estimated the linear regression coefficient slopes by sex and country with accompanying 95% CI. A robust regression analysis was also performed, which lessens the influence of outliers on the regression coefficient estimates. Estimates were almost identical to those in the least-squares linear regression.

The self-reported silhouette showcards were assessed for accuracy in predicting widely used dichotomized anthropometric adiposity measures, e.g., overweight and obesity (BMI ≥ 25 kg/m^2^) or obesity alone (BMI ≥ 30 kg/m^2^), elevated waist circumference (≥ 94 cm in men, ≥ 80 cm in women) and elevated waist-to-height ratio (WHtR > 0.5) using sex and country-specific receiver-operator curve (ROC) analysis [26]. In line with previous studies, we used the area under the curve (AUC, i.e., the c-statistic) and sensitivity and specificity associated with different cutoffs of the silhouettes to predict these dichotomous adiposity categories.

All statistical analyses were performed using STATA SE 12 (StataCorp, College Station, TX, US).

## Results

### Demographics

Table 1 shows the main characteristics of the 751 participants from the three countries. The study sample consisted of men and women aged 20–68 years old. Approximately 66% of the whole sample identified as female. Mean age for men ranged from highest in the US (47.1 years) and significantly lower in Seychelles men (45.2) (*p* < 0.05). Women’s mean age was also highest in the US (45.3) and significantly lower in Ghanaian women (41.4).

**Table 1:** Characteristics of participants in the United States, Seychelles, and Ghana.

	United States		Seychelles		Ghana	
	Men (*N* = 88)	Women (*N* = 177)	Men (*N* = 100)	Women (*N* = 183)	Men (*N* = 67)	Women (*N* = 136)
**Age (years)**	47.1 [45.9–48.3]	45.3 [44.3–46.2]	45.2 [44.2–46.2]*	44.3 [43.4–45.2]	45.6 [43.4–47.7]	41.4 [40.0–42.8]*
**Height (cm)**	174.8 [173.4–176.2]	164.8 [163.7–165.8]	173.6 [172.4–174.7]	162.1 [161.1–163.2]*	167.9 [166.3–169.6] *	159.1 [158.2–160.0]*
**Weight (kg)**	88.2 [83.6–92.9]	97.3 [93.8–100.8]	85.3 [82.0–88.5]	80.3 [77.4–83.1]*	67.3 [65.0–69.5]*	71.9 [69.5–74.4]*
**BMI (kg/m^2^)**	28.9 [27.4–30.4]	35.8 [34.5–37.0]	28.3 [27.3–29.3]	30.5 [29.5–31.6]*	23.9 [23.1–24.7]*	28.5 [27.5–29.4]*
**Waist circumference (cm)**	100.0 [96.1–103.6]	109.3 [106.8–111.9]	95.8 [93.6–98.1]*	95.6 [93.6–97.5]*	86.3 [83.9–88.7]*	96.1 [94.0–98.1]*
**Waist-to-height ratio**	0.57 [0.55–0.59]	0.66 [0.65–0.68]	0.55 [0.54–0.57]	0.59 [0.58–0.60]*	0.52 [0.50–0.53]*	0.6 [0.59–0.62]*
**Perceived silhouette (1–9)**	4.2 [3.9–4.6]	6.2 [5.9–6.4]	4.5 [4.3–4.8]	5.4 [5.1–5.6]*	4.1 [3.6–4.5]	5.5 [5.1–5.8]*
**Anthropometric adiposity measures, %**						
**Underweight**	2.3 [0.0–5.4]	0.6 [0.0–1.7]	2 [0.0–4.8]	0.5 [0.0–1.6]	1.5 [0.0–4.4]	1.5 [0.0–3.5]
(BMI < 18.5 kg/m^2^)						
**Normal weight**	26.1 [16.9–35.4]	6.2 [2.6–9.8]	26 [17.3–34.7]	21.9 [15.8–27.9]*	61.2 [49.4–73.0]*	27.9 [20.4–35.5]*
(BMI 18.5–24.9 kg/m^2^)						
**Overweight**	36.4 [26.2–46.5]	19.2 [13.4–25.0]	41 [31.3–50.7]	29 [22.4–35.6]*	34.3 [22.8–45.8]	35.3 [27.2–43.4]*
(BMI 25–29.9 kg/m^2^)						
**Obese**	35.2 [25.1–45.3]	74 [67.5–80.5]	31 [21.8–40.2]	48.6 [41.4–55.9]*	3 [0.0–7.1]*	36 [27.9–44.1]*
(BMI ≥ 30 kg/m^2^)						
**Central obesity**	55.7 [45.1–66.3]	96.6 [93.9–99.3]	54 [44.1–63.9]	88.5 [83.9–93.2]*	29.9 [18.6–41.1]*	92.6 [88.2–97.1]
(WC ≥ 94 cm M; ≥ 80 cm W)						
**Elevated waist-to-height ratio** (> 0.5)	73.9 [64.5–83.2]	94.9 [91.6–98.2]	79 [70.9–87.1]	83.6 [78.2–89.0]*	44.8 [32.6–57.0]*	90.4 [85.4–95.4]

Data are presented as mean or proportions [95% confidence intervals]. BMI: body mass index; WC: waist circumference.

* *p* < 0.05 compared to United States.

### Anthropometric and adiposity measures

All measures of size, including height, weight, and adiposity, were significantly higher in the US, intermediate in Seychelles, and lowest in Ghana, and was higher in women than men in each country. For example, mean BMI (kg/m^2^) in men/women was 28.9/35.8 in the US, 28.3/30.5 in Seychelles, and 23.9/28.5 in Ghana (Table 1), with similar trends for WC and WHtR. The mean values of anthropometric measures and the prevalence of the anthropometric adiposity measures were lowest in men from Ghana. The prevalence (%) of obesity in men/women (BMI ≥ 30) followed a similar decreasing trend: 35.2/74.0 in the US, 31.0/48.6 in Seychelles, and 3.0/36.0 in Ghana (Table 1). The prevalence of elevated WC exceeded 88.5% in women from all three countries. Men in the US and Seychelles had an intermediate prevalence of elevated WC (55.7% and 54.0%, respectively) versus Ghanaian men (29.9%). Finally, the prevalence of elevated WHtR (> 0.5) exceeded 70% in men and women from all countries, except in men from Ghana (44.6%) (Table 1). The different adiposity markers used in this study inter-correlated quite strongly in men and women. Correlation coefficients were 0.91 for men/0.77 for women for the association between BMI and WC, 0.94/0.80 for the association between BMI and WHtR, and 0.96/0.95 for the association between WC and WHtR in the US; 0.89/0.91, 0.91/0.92, and 0.94/0.95 in Seychelles; and 0.88/0.91, 0.90/0.90, and 0.92/0.96 in Ghana.

### Correlations between silhouette showcards and continuous anthropometric measures

Table 2 shows Spearman’s correlation coefficients between the perceived self-reported silhouette rankings with BMI, WC, and WHtR, by country and sex. These coefficients ranged between 0.71 and 0.80 in men and women in all countries, except in men in Ghana (0.55-0-58) (*p* < 0.001 for all coefficients).

**Table 2:** Spearman correlation coefficients (r) between self-reported silhouette ranking and continuous anthropometric measures in men and women from the US, Seychelles, and Ghana.

	Men [95% CI]	Women [95% CI]
**United States, *N***	*88*	*177*
**BMI (kg/m^2^)**	0.77 [0.66–0.87]	0.79 [0.73–0.85]
**WC (cm)**	0.72 [0.60–0.83]	0.74 [0.67–0.82]
**Waist-to-height ratio**	0.75 [0.64–0.86]	0.75 [0.68–0.82]
**Seychelles, *N***	*100*	*183*
**BMI (kg/m^2^)**	0.78 [0.71–0.87]	0.80 [0.74–0.85]
**WC (cm)**	0.76 [0.66–0.86]	0.77 [0.71–0.84]
**Waist-to-height ratio**	0.79 [0.70–0.88]	0.76 [0.70–0.83]
**Ghana, *N***	*67*	*136*
**BMI (kg/m^2^)**	0.56 [0.39–0.73]	0.74 [0.67–0.82]
**WC (cm)**	0.55 [0.37–0.73]	0.73 [0.65–0.82]
**Waist-to-height ratio**	0.58 [0.41–0.75]	0.71 [0.63–0.80]

BMI: body mass index (kg/m^2^); WC: waist circumference (cm); CI: confidence intervals; *N*: sample size. The *P*-value for all correlations is <0.001.

### Relationship between silhouette ranking and measured BMI

Table 3 shows a graded increase in mean BMI according to silhouette ranking by sex and country. The table also depicts the least-squares linear regression coefficients by sex and country between participants’ measured BMI and the self-reported silhouettes. Regression coefficients (i.e., slopes of the regression lines) were higher in women than men in all three countries. However, regression coefficients were significantly lower in Ghana than in the other two countries for both men and women. For example, in the US and Seychelles, an increase in 1 silhouette unit was associated with an increase of 3.05–3.75 BMI units (kg/m^2^) but only 1.15–2.06 BMI units in Ghana. Nearly identical trends were observed for WC and WHtR (S1 and S2 Tables).

**Table 3:** Mean BMI by silhouette number, country, and sex.

	United States (*N* = 265)				Seychelles (*N* = 283)				Ghana (*N* = 203)			
	*N* (all)	Men	Women	All	*N* (all)	Men	Women	All	*N* (all)	Men	Women	All
		Mean [95% CI]	Mean [95% CI]	Mean [95% CI]		Mean [95% CI]	Mean [95% CI]	Mean [95% CI]		Mean [95% CI]	Mean [95% CI]	Mean [95% CI]
**Silhouette 1**	4	20 [0–40.4]	21 [15.3–27.4]	20 [15.6–25.3]	0	*	*	*	8	22 [19.9–23.3]	21 [15.8–26.3]	21 [19.4–23.2]
**Silhouette 2**	14	23 [20.6–25.4	25 [19.4–29.8]	23 [21.2–25.4]	20	21 [19.1–23.4]	22 [20.6–22.3]	21 [20.5–22.3]	16	21 [19.6–21.8]	22 [19.6–23.8]	21 [20.1–22.4]
**Silhouette 3**	24	25 [23.1–26.2]	25 [23.1–26.4]	25 [23.4–25.9]	21	24 [22.3–26.0]	23 [20.7–25.2]	24 [22.1–24.9]	28	23 [21.2–23.9]	24 [21.4–25.8]	23 [21.9–24.2]
**Silhouette 4**	40	28 [26.3–29.1]	28 [25.7–29.5]	28 [26.5–28.8]	67	26 [25.0–27.0]	26 [24.8–26.9]	26 [25.2–26.6]	26	24 [21.8–25.9]	25 [24.1–26.2]	25 [23.6–25.6]
**Silhouette 5**	43	30 [27.4–32.0]	31 [29.5–32.2]	31 [29.3–31.7]	63	30 [28.5–31.2]	29 [27.5–30.5]	29 [28.3–30.4]	38	25 [23.0–26.5]	27 [25.2–28.1]	26 [24.7–26.9]
**Silhouette 6**	57	33 [29.7–35.6]	35 [33.5–36.1]	34 [33.2–35.6]	55	33 [30.6–35.6]	31 [30.1–32.7]	32 [30.7–33.0]	37	26 [23.1–29.1]	29 [27.6–31.0]	29 [27.1–30.1]
**Silhouette 7**	40	41 [35.1–47.1]	38 [36.3–40.5]	39 [36.8–40.8]	38	36 [34.1–38.4]	35 [33.4–36.8]	35 [33.8–36.7]	27	29 [24.5–32.7]	32 [30.2–34.0]	32 [29.9–33.3]
**Silhouette 8**	25	48 [0–126]	41 [38.6–43.8]	42 [39.0–44.5]	17	37 [*]	43 [38.7–47.2]	43 [38.6–46.6]	17	26 [*]	33 [30.3–35.7]	33 [29.9–35.3]
**Silhouette 9**	18	55 [*]	50 [46.6–53.4]	50 [46.7–53.7]	2	*	45 [29.8–59.5]	45 [29.8–59.5]	6	*	38 [29.4–46.5]	38 [29.4–46.5]
**Reg coeff**	265	3.51 [2.98–4.04]	3.75 [3.28–4.21]	3.65 [3.34–3.97]	238	3.05 [2.56–3.54]	3.34 [2.94–3.74]	3.23 [2.93–3.54]	203	1.15 [0.73–1.56]	2.06 [1.71–2.41]	1.99 [1.72–2.26]

BMI: body mass index; CI: Confidence interval; Reg coeff: Linear Regression coefficients.

* indicates no data.

Mean BMI’s rounded to the nearest whole number.

### Self-reported silhouette as a detector of overweight or obesity

Based on the graded increase in mean BMI presented in Table 3, silhouettes 4, 5, 6, and 7 were used to attempt to detect men and women who were overweight and obese or only obese. Table 4 shows that the predictive value decreased with increasing silhouette ranking. For silhouette ≥ 4, the sensitivity to predict BMI ≥ 25 ranged between 91.4–96.7% and was 98.8–100% to predict BMI ≥ 30, while for silhouette ≥ 7, sensitivity ranged from 26.9–41.2% to predict BMI ≥ 25 and was 45–68.6% to predict BMI ≥ 30. For silhouette ≥ 4, the specificity of predicting BMI ≥ 25 ranged between 47.9–68.8% and was 24.5–38.8% to predict BMI ≥ 30. For silhouette ≥ 7, specificity ranged from 98.8–100% to predict BMI ≥ 25 and was 90.1–98.2% to predict BMI ≥ 30.

**Table 4:** Sensitivity and specificity of self-selected silhouette ratings to detect overweight and obesity, or obese only in US, Seychelles, and Ghana.

		Overweight and Obese		Obesity	
		Sensitivity (%)	Specificity (%)	Sensitivity (%)	Specificity (%)
**Silhouette ≥ 4**	US	91.4	68.8	98.8	38.8
	Seychelles	96.7	47.9	99.2	24.5
	Ghana	93.3	52.4	100.0	34.2
**Silhouette ≥ 5**	US	77.3	90.6	92.0	67.0
	Seychelles	77.8	85.9	95.0	62.6
	Ghana	84.9	71.4	100.0	51.3
**Silhouette ≥ 6**	US	59.7	96.9	79.0	88.4
	Seychelles	52.4	98.6	75.0	86.5
	Ghana	66.4	90.5	92.2	73.7
**Silhouette ≥ 7**	US	35.6	100.0	49.4	97.1
	Seychelles	26.9	100.0	45	98.2
	Ghana	41.2	98.8	68.6	90.1

Overweight and Obese: BMI ≥ 25 kg/m^2^; Obesity: BMI ≥30 kg/m^2^; US: United States.

**Fig 2:**
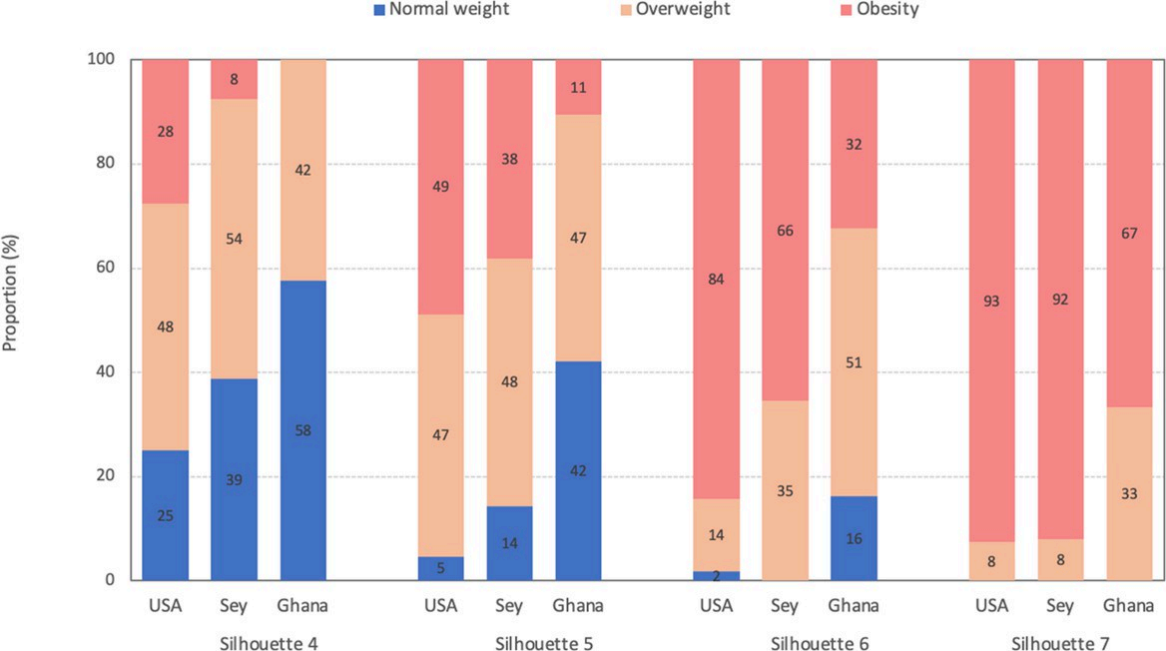
Proportion with normal weight, overweight, and obese within each silhouette category in the US, Seychelles, and Ghana. N weight: normal weight (BMI 18.5–24.9 kg/m^2^); Overweight (BMI 25.0–29.9 kg/m^2^); Obese (BMI ≥ 30 kg/m^2^); Sey: Seychelles.

Fig 2 depicts the proportion of participants categorized as normal weight, overweight, or obese for the middle four silhouettes (4–7). Silhouettes 4 and 5 captured the largest proportion of overweight participants in the US, and Seychelles, while silhouettes 5 and 6 captured the most overweight participants in Ghana. When assessing obesity status, silhouette 7 in the US, Seychelles, and Ghana captured most obese participants.

### Performance between silhouette ranking to BMI, waist circumference, and waist-to-height ratio in predicting adiposity

Table 5 shows the sex and country-specific AUCs (i.e., c-statistic) of silhouette ranking to predict overweight and obesity status (BMI ≥ 25 kg/m^2^) or obesity alone (BMI ≥ 30 kg/m^2^). AUCs ranged between 0.79 and 0.92 in men and between 0.87 and 0.97 in women, with minor differences by sex or country. Similar AUC values were found for silhouette ranking to predict elevated WC and WHtR.

**Table 5:** Performance of self-reported silhouette ranking to predict overweight and obese BMI, elevated WC, and elevated WHtR in the US, Seychelles, and Ghana.

		Overweight and Obese	Obesity	Elevated WC	Elevated WHtR
Country	Sex	AUC [95% CI]	AUC	AUC	AUC
**US**	M	0.79 [0.70–0.89]	0.88 [0.81–0.95]	0.82 [0.74–0.90]	0.84 [0.75–0.93]
	W	0.97 [0.95–0.99]	0.88 [0.83–0.94]	0.96 [0.93–1.00]	0.94 [0.89–0.98]
	All	0.91 [0.86–0.95]	0.91 [0.87–0.94]	0.91 [0.88–0.95]	0.91 [0.88–0.95]
**Seychelles**	M	0.87 [0.80–0.93]	0.86 [0.82–0.95]	0.85 [0.77–0.92]	0.88 [0.80–0.96]
	W	0.91 [0.87–0.95]	0.89 [0.85–0.94]	0.91 [0.84–0.97]	0.89 [0.84–0.94]
	All	0.89 [0.86–0.93]	0.89 [0.86–0.93]	0.87 [0.83–0.92]	0.88 [0.84–0.93]
**Ghana**	M	0.85 [0.76–0.94]	0.92 [0.83–1.00]	0.77 [0.65–0.88]	0.83 [0.73–0.92]
	W	0.87 [0.80–0.93]	0.87 [0.82–0.93]	0.88 [0.79–0.96]	0.86 [0.77–0.95]
	All	0.87 [0.82–0.92]	0.90 [0.86–0.94]	0.81 [0.75–0.87]	0.84 [0.79–0.90]

M: men; W: women; BMI: body mass index (kg/m^2^); WC: waist circumference (cm); WHtR: waist-to-height ratio; AUC: area under the curve; 95% CI: 95% confidence interval. Overweight and obese: BMI ≥ 25 kg/m^2^; Obesity: BMI ≥ 30 kg/m^2^; Elevated WC: M ≥ 94 cm; W ≥ 80 cm; Elevated WHtR > 0.5.

## Discussion

This study continues the foundation established by Pulvers and colleagues in creating the silhouette showcards and subsequent validation in populations of African-origin [25–27]. However, our study is the first to use Pulvers silhouette showcards across different populations using the same methodology. We showed that the silhouette showcards have a strong relationship to measured anthropometrics, can detect overweight and obesity, and might be a helpful tool for predicting adiposity measures such as elevated BMI, WC, and WHtR in different populations of both adult men and women of mainly African-origin. However, the relationship between silhouettes and adiposity measures differed according to the country, and no universal silhouette cutoff can predict overweight or obesity across populations. Overall, our data suggest that silhouettes may be a useful tool to predict actual anthropometric and adiposity measures, conditional to adequate calibration for a specific population.

BMI and other anthropometrics correlated strongly with silhouette ranking in all populations. However, the magnitude of the linear regression coefficients between silhouette ranking and actual anthropometrics differed between the three countries in this study. For example, an increase of 1 silhouette unit was associated with an increase of 3–4 BMI units (kg/m^2^) in the US and Seychelles but only 1–2 BMI units in Ghana (Table 3). This difference suggests varying perceptions of one’s body shape, possibly according to mean population BMI. One may speculate that in the US and Seychelles, where mean population BMI is high, individuals with adiposity are more inclined to view a large body shape as normal compared to populations (e.g., Ghana) where mean population BMI is lower. Again, this altered view suggests that silhouette showcards need to be specific (i.e., calibrated) to different populations when used for predicting individuals’ actual anthropometrics. From a prevention perspective, the differences in perceptions of one’s body size across populations may suggest larger tolerance for larger body shapes in populations with high obesity prevalence. Overall, this underlies that silhouettes can have a role in assessing body size in populations when direct measurements cannot be made (i.e., for surveillance purposes, as evaluated in this study), but may also be used to assess perceptions and attitudes of people for weight control programs.

Our data shows that when self-reported silhouette ranking and continuous anthropometric measures correlate between men and women within a population, it is likely that the same predictive linear regression models can be used. Previous studies using different silhouette showcards have shown, Spearman correlation coefficients for the relationship between silhouettes and BMI (kg/m^2^ per silhouette unit) were, for example, 0.73 for men and 0.81 for women among white Americans (with a mean BMI of 25.5 kg/m^2^ in men and 24.1 kg/m^2^ in women) and 0.73 for men and 0.80 in Japanese women (with mean BMI of 23.3 kg/m^2^ in men and 21.5 kg/m^2^ in women) and 0.80 for men and 0.81 for women in Seychelles (with mean BMI of 26.4 kg/m^2^ in men and 29.3 kg/m^2^ in women) [21, 22, 26]. Our correlation coefficients for the US and Seychelles were like these previous studies (Table 2). Given that the regression coefficients were similar between men and women within their respective population, it can be proposed that a linear regression model can be used for both sexes to calibrate the association between silhouettes and BMI (or other adiposity markers) within the same population. This assumption can stand if the silhouette and anthropometric correlations between sexes are similar, like in the US and Seychelles. Inversely, as our data in Ghana suggest, different predictive regression models may need to be developed in men and women when Spearman correlation coefficients markedly differ between sexes (correlation coefficient 0.55–0.58 in men compared to 0.71–0.74 in women) in the same population (Table 2). Differences in the regression coefficients between silhouettes and BMI (and other adiposity markers) may also partly depend on different mean population BMI and sex-specific perceptions of body shape, and these questions necessitate further studies.

Different silhouette cutoffs detected obesity differently in the three countries. While the BMI categories for each silhouette rank showed a large dispersion, there were apparent differences in the country’s distribution pattern (Fig 2). In addition, each silhouette also had varying sensitivities and specificity between countries for detecting overweight or obesity status (Table 4). This variation suggests that no universal silhouette cutoff can be used for detecting overweight or obesity status.

The country and sex-specific associations between silhouettes and adiposity measures were similar for BMI, WC, and WHtR. This relationship is not unexpected as BMI, WC, and WHtR quite strongly and similarly inter-correlate with each other, e.g., correlation coefficients of 0.77 to 0.96 in our study, which is consistent with correlations found in other studies [42]. However, the associations between silhouettes and BMI, WC, and WHtR are still not extremely strong, implying that silhouettes would not be a reliable tool to predict adiposity at the individual level (sensitivity and specificity are not optimal). However, they can be helpful when assessing adiposity levels (e.g., the prevalence of obesity, mean BMI) at a population level, conditional on appropriate calibration in a specific population. More generally, our data suggest that a subjective two-dimensional pictorial body size assessment (silhouette drawings) can be a valuable tool for predicting a volumetric dimension (adiposity), at least at the population level.

This study’s main strength was using the identical methodology in the three countries, allowing us to directly compare three populations that differed largely according to mean adiposity levels and socioeconomic development stages. However, the study also has limitations. First, although the study was designed to include participants of African-origin in all sites, to control for ethnic differences, persons from mixed origins were also included in varying but small proportions, particularly in Seychelles. Second, the study included adults aged 20–68, and the findings may not necessarily extend to older or younger individuals. Third, Pulvers’ silhouette tool presents body size silhouettes from thinnest to heaviest, possibly leading to reporting bias. Future studies should examine if presenting the silhouettes in random order would gather different results. Fourth, survey administrators presented silhouettes to the participants; further studies should assess if results would differ if participants had assessed their silhouettes in the absence of assisting personnel. Finally, our analysis, according to sex, was limited because of the limited sample size.

## Conclusions

This study supports the utility of Pulvers’ silhouette showcards as a useful tool to predict anthropometric and adiposity measures in different populations and in settings where body size cannot be measured directly. However, no universal silhouette cutoff can be used to detect overweight or obesity status, and caution should be used to ensure adequate adjustment (i.e., calibration) for the associations between silhouette ranking and actual adiposity measures between sexes and countries. In addition, further assessment should be done to examine sex-specific differences in body perception and cultural ideals in body size across the epidemiological transition.

## Supporting information

S1 Table Mean waist circumference (cm) by silhouette number, country, and sex. CI: 95% confidence interval; Reg coeff: Linear Regression coefficients. * indicates no data. Mean waist circumference rounded to the nearest whole number.
(PDF)

S2 Table Mean waist-to-height ratio by silhouette number, country, and sex. CI: 95% confidence interval; Reg coeff: Linear Regression coefficients. * indicates no data.
(PDF)

S1 Data Dataset used and analyzed during the current study. (XLSX)
