# Supplementary material for: Utility of silhouette showcards to assess adiposity in three countries across the epidemiological transition
Source: PLOS Glob Public Health. 2022 May 19;2(5):e0000127. doi: 10.1371/journal.pgph.0000127 (PMC10021870; doi:10.1371/journal.pgph.0000127)
Supplement: S1 Table — CI: 95% confidence interval; Reg coeff: Linear Regression coefficients. * indicates no data. Mean waist circumference rounded to the nearest whole number. (PDF) [file pgph.0000127.s001.pdf]

Supplemental Table 1. Mean waist circumference (cm) by silhouette number, country, and sex

	United States (*N*=265)				Seychelles (*N*=283)				Ghana (*N*=203)			
	***N* (all)**	**Men**	**Women**	**All**	***N* (all)**	**Men**	**Women**	**All**	***N* (all)**	**Men**	**Women**	**All**
		**Mean [95% CI]**	**Mean [95% CI]**	**Mean [95% CI]**		**Mean [95% CI]**	**Mean [95% CI]**	**Mean [95% CI]**		**Mean [95% CI]**	**Mean [95% CI]**	**Mean [95% CI]**
**Silhouette 1**	4	79 [59.4-97.6]	82 [51.7-113.0]	80 [65.3-95.6]	0	*	*	*	8	78 [71.1-85.5]	81 [75.5-85.5]	79 [75.1-83.2]
**Silhouette 2**	14	86 [80.9-91.7]	85 [73.0-96.9]	86 [81.3-90.8]	20	83 [77.8-88.8]	78 [75.0-81.5]	80 [77.1-83.3]	16	77 [73.2-81.5]	82 [76.8-86.4]	80 [76.4-83.1]
**Silhouette 3**	24	89 [84.8-93.1]	86 [76.7-95.8]	88 [84.6-92.2]	21	85 [82.2-88.6]	79 [75.1-83.1]	82 [79.2-85.0]	28	83 [79.6-86.2]	86 [81.7-90.3]	84 [81.5-86.9]
**Silhouette 4**	40	97 [92.2-100.9]	92 [87.0-96.2]	94 [91.1-97.6]	67	91 [89.2-93.0]	88 [85.0-89.9]	89 [87.7-90.9]	26	87 [81.0-92.8]	88 [85.8-90.1]	88 [84.8-90.2]
**Silhouette 5**	43	103 [97.0-109.2]	100 [95.7-103.5]	101 [97.4-104.0]	63	98 [94.8-100.7]	93 [90.5-96.0]	95 [93.1-97.3]	38	89 [84.1-93.8]	92 [88.1-95.5]	91 [87.6-93.6]
**Silhouette 6**	57	109 [101.0-116.1]	108 [105.4-111.2]	108 [105.6-111.1]	55	108 [102.0-113.8]	97 [94.6-99.3]	100 [97.2-102.6]	37	90 [83.8-97.0]	99 [95.4-102.8]	97 [93.8-100.6]
**Silhouette 7**	40	131 [117.6-144.0]	116 [111.4-121.1]	118 [113.7-123.2]	38	114 [107.4-121.1]	105 [101.3-108.7]	107 [103.0-109.9]	27	102 [93.6-111.0]	103 [99.9-106.5]	103 [100.1-106.1]
**Silhouette 8**	25	137 [122.2-150.8]	120 [115.5-124.5]	121 [116.7-126.0]	17	111 [*]	116 [109.4-123.4]	116 [109.5-122.7]	17	92 [*]	107 [100.5-113.2]	106 [99.7-112.2]
**Silhouette 9**	18	163 [*]	134 [125.4-142.1]	153 [126.9-144.2]	2	*	124 [0-340.9]	124 [0.0-340.9]	6	*	114 [101.4-126.1]	114 [101.4-126.1]
**Reg coeff**	265	8.3 [6.88-9.73]	7.34 [6.33-8.35]	7.05 [6.32-7.79]	238	6.51 [5.36-7.65]	6.24 [5.46-7.02]	5.89 [5.24-6.53]	203	3.37 [2.16-4.59]	4.39 [3.66-5.12]	4.45 [3.85-5.04]

Notes: CI: confidence interval; Reg coeff: Linear Regression coefficients. * indicates no data. Mean waist circumference rounded to the nearest whole number.
