# Supplementary material for: Utility of silhouette showcards to assess adiposity in three countries across the epidemiological transition
Source: PLOS Glob Public Health. 2022 May 19;2(5):e0000127. doi: 10.1371/journal.pgph.0000127 (PMC10021870; doi:10.1371/journal.pgph.0000127)
Supplement: S2 Table — CI: 95% confidence interval; Reg coeff: Linear Regression coefficients. * indicates no data. (PDF) [file pgph.0000127.s002.pdf]

Supplemental Table 2. Mean waist-to-height ratio by silhouette number, country, and sex

	United States (*N*=265)				Seychelles (*N*=283)				Ghana (*N*=203)			
	N (all)	Men	Women	All	*N* (all)	Men	Women	All	*N* (all)	Men	Women	All
		**Mean** [95% CI]	**Mean** [95% CI]	**Mean** [95% CI]		**Mean** [95% CI]	**Mean** [95% CI]	**Mean** [95% CI]		**Mean** [95% CI]	**Mean** [95% CI]	**Mean** [95% CI]
**Silhouette 1**	4	0.44 [0-0.88]	0.51 [-0.25-1.3]	0.47 [0.36-0.59]	0	*			8	0.46 [0.44-0.47]	0.50 [0.43-0.56]	0.48 [0.45-0.51]
**Silhouette 2**	14	0.48 [0.45-0.52]	0.53 [0.33-0.72]	0.50 [0.46-0.53]	20	0.48 [0.43-0.51]	0.48 [0.46-0.51]	0.48 [0.46-0.50]	16	0.46 [0.44-0.48]	0.51 [0.47-0.55]	0.49 [0.46-0.51]
**Silhouette 3**	24	0.51 [0.48-0.53]	0.53 [0.46-0.60]	0.51 [0.49-0.53]	21	0.49 [0.46-0.51]	0.50 [0.46-0.53]	0.49 [0.47-0.51]	28	0.49 [0.47-0.51]	0.54 [0.51-0.57]	0.51 [0.49-0.53]
**Silhouette 4**	40	0.56 [0.53-0.58]	0.56 [0.53-0.58]	0.56 [0.54-0.57]	67	0.53 [0.52-0.54]	0.54 [0.52-0.56]	0.53 [0.52-0.54]	26	0.53 [0.49-0.56]	0.55 [0.53-0.56]	0.54 [0.52-0.56]
**Silhouette 5**	43	0.59 [0.55-0.63]	0.61 [0.58-0.63]	0.60 [0.58-0.62]	63	0.57 [0.55-0.58]	0.57 [0.55-0.59]	0.57 [0.56-0.58]	38	0.53 [0.50-0.57]	0.58 [0.55-0.60]	0.56 [0.54-0.58]
**Silhouette 6**	57	0.62 [0.58-0.67]	0.66 [0.64-0.67]	0.65 [0.63-0.67]	55	0.61 [0.58-0.65]	0.60 [0.59-0.62]	0.60 [0.59-0.62]	37	0.54 [0.49-0.59]	0.63 [0.60-0.65]	0.61 [0.58-0.63]
**Silhouette 7**	40	0.75 [0.67-0.83]	0.71 [0.68-0.74]	0.71 [0.69-0.74]	38	0.66 [0.63-0.69]	0.64 [0.62-0.66]	0.65 [0.63-0.66]	27	0.62 [0.54-0.69]	0.65 [0.60-0.68]	0.65 [0.63-0.67]
**Silhouette 8**	25	0.79 [0.086-1.5]	0.73 [0.70-0.76]	0.73 [0.70-0.76]	17	0.63 [*]	0.73 [0.68-0.78]	0.73 [0.68-0.77]	17	0.54 [*]	0.67 [0.63-0.71]	0.66 [0.62-0.70]
**Silhouette 9**	18	0.98 [*]	0.81 [0.76-0.86]	0.82 [0.77-0.87]	2	*	0.75 [0.24-1.3]	0.75 [0.24-1.26]	6	*	0.72 [0.61-0.82]	0.72 [0.61-0.82]
**Reg coeff**	265	0.050 [0.042-0.058]	0.043 [0.037-0.050]	0.046 [0.0418-0.0504]	238	0.038 [0.032-0.044]	0.039 [0.034-0.044]	0.039 [0.0351-0.0426]	203	0.022 [0.014-0.030]	0.028 [0.023-0.033]	0.309 [0.0266-0.0351]

Notes: CI: confidence interval; Reg coeff: Linear Regression coefficients. * indicates no data.
